# Impact of Release Rates on the Effectiveness of Augmentative Biological Control Agents

**DOI:** 10.1673/031.007.1501

**Published:** 2007-03-19

**Authors:** David W. Crowder

**Affiliations:** Department of Entomology, University of Arizona, Tucson, AZ

## Abstract

To access the effect of augmentative biological control agents, 31 articles were reviewed that investigated the impact of release rates of 35 augmentative biological control agents on the control of 42 arthropod pests. In 64% of the cases, the release rate of the biological control agent did not significantly affect the density or mortality of the pest insect. Results where similar when parasitoidsor predators were utilized as the natural enemy. Within any order of natural enemy, there were more cases where release rates did not affect augmentative biological control than cases where release rates were significant. There were more cases in which release rates did not affect augmentative biological control when pests were from the orders Hemiptera, Acari, or Diptera, but not with pests from the order Lepidoptera. In most cases, there was an optimal release rate that produced effective control of a pest species. This was especially true when predators were used as a biological control agent. Increasing the release rate above the optimal rate did not improve control of the pest and thus would be economically detrimental. Lower release rates were of ten optimal when biological control was used in conjunction with insecticides. In many cases, the timing and method of biological control applications were more significant factors impacting the effectiveness of biological control than the release rate. Additional factors that may limit the relative impact of release rates include natural enemy fecundity, establishment rates, prey availability, dispersal, and cannibalism.

## Introduction

Biological control is often viewed as a promising alternative or complement to pesticides in integrated pest management programs (McDougall and Mills 1997). The successes and failures of biological control have been extensively reviewed (DeBach 1964; Huffaker and Messenger 1976; DeBach and Rosen 1991; van Driesche and Bellows 1996; Collier and van Steenwyk 2004; Stlling and Cornelissen 2005). Factors that can influence the effectiveness of biological control agents include agent specificity (generalist or specialist), the type of agent (predator, parasitoid, or pathogen), the timing and number of releases, the method of release, synchrony of the natural enemy with the host, field conditions, and release rate (DeBach 1964; Beirne 1975; Huffaker and Messenger 1976; DeBach and Rosen 1991; van Driesche and Bellows 1996; Lester et al. 1999; Collier and van Steenwyk 2004; Stiling and Cornelissen 2005).

The purpose of this paper is to assess the relative impact of release rates on the effectiveness of augmentative biological control. Augmentative, or inundative, biological control is the release of large numbers of natural enemies to augment natural enemy populations or inundate pest populations with natural enemies (Collier and van Steenwyk 2004). The analysis was based on a review of articles in which the effectiveness of augmentative biological control agents as a function of release rates were measured. The effect of release rates on the successful implementation of augmentative biological control was assessed when parasitoids and predators were utilized as biological control agents. In addition, the relative impact of release rates were compared to factors such as the method and timing of releases and when pesticides were used in conjunction with biological control agents.

It was found that increasing the number of biological control agents released into an environment did not always increase the level of pest control. Releasing a greater number of biological control agents increased the cost of implementing biological control (van Driesche et al. 2001; 2002; Collier and van Steenwyk 2004). Thus, if increasing the release rate does not improve control, releasing fewer natural enemies would result in more efficient and economically beneficial augmentative biological control.

## Materials and Methods

### Literature review

Two databases (BIOSIS Previews and Web of Science), were searched using the key words: “biological control” and either “release rate”, “release density”, or “number released.” Thirty-nine studies of augmentative biological control were identified where a biological control agent was released at various densities and the effectiveness of the agent as a function of release rates was measured.

All studies included in the review were required to have used an appropriate experimental design (Collier and van Steenwyk 2004), which included: (1) treatment(s) in which natural enemies were released at two or more release rates in replicated experimental units and (2) a control treatment in which no natural enemies were released in other replicated experimental units. Studies were excluded from the review if they used an inappropriate experimental design. Studies were also excluded from the review if the natural enemy had no significant effect on the target pest at any release rate compared to the control treatment. Thus, in all studies included in the analysis, the augmentative biological control agent was effective in suppressing the target pest with at least one release rate.

Of the 39 reviewed studies, 31 met the conditions for inclusion. These studies analyzed the impact of 35 biological control agents on 42 pests with various release rates (Table 1). A separate analysis was done for cases in which parasitoids or predators were utilized as the biological control agent. In addition, the effect of release rates was examined when targeted pests were grouped by order. In this analysis, results are presented by pest order regardless of whether the biological control agent utilized was a parasitoid or predator.

The studies varied in the effects measured (Table 1). Despite this, the same approach was used for evaluating whether release rates significantly affected augmentative biological control across all studies. This approach was based on whether the author(s) indicated that pest populations or damage were suppressed below some specified target density or damage level, or that parasitism rates increased above a target level, in the highest density release treatments but not in lower density release treatments. A similar approach has been used in other review studies of augmentative biological control (Collier and van Steenwyk 2004).

**Table 1a.**
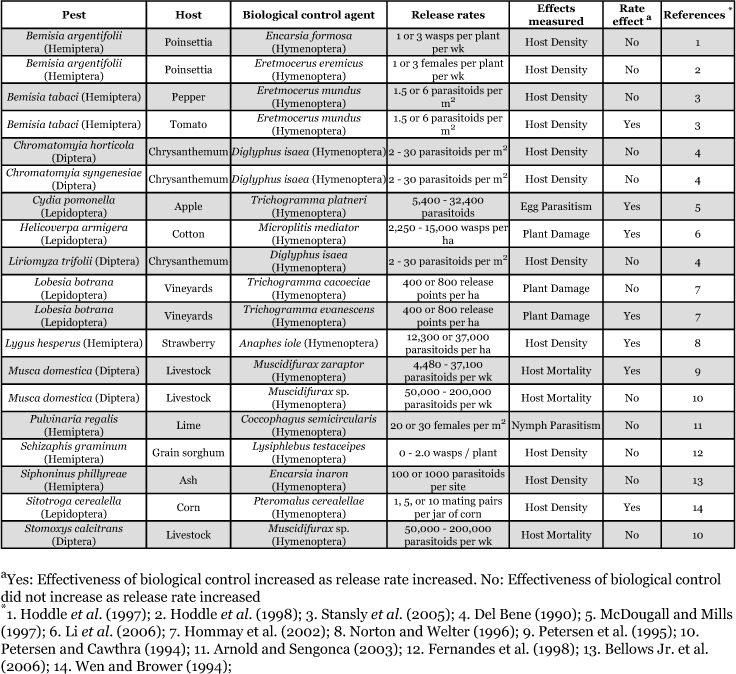
Impact of release rates of biological control agents on selected pests (Parasitoids)

### Comparison of parasitoids and predators

Fifteen of the biological control agents (43%) were parasitoids. All of the parasitoids were from the order Hymenoptera (Table 1). Nineteen pests (45%) were targeted with parasitoids. Of the pests targeted with parasitoids, the most common order was Hemiptera (8 pests; 42%), followed by Diptera (6 pests; 32%), and Lepidoptera (5 pests; 26%) (Table 1).

Twenty of the biological control agents (57%) were predators. The order most commonly used as a predatory biological control agent was Acari (11 agents, 55%), followed by Hemiptera (6 agents, 30%), Coleoptera (2 agents, 10%) and Neuroptera (1 agent, 5%) (Table 1). Twenty-three pests (55%) were targeted with predators. The most common order targeted with predators was Acari (11 pests; 48%), followed by Hemiptera (7 pests, 30%), Coleoptera (2 pests, 9%), Thysanoptera (2 pests, 9%), and Lepidoptera (1 pest, 4%) (Table 1).

### Impact of release rates relative to other factors

Several studies analyzed the impact of release rates compared to other factors that impacted the effectiveness of an augmentative biological control agent. Five studies compared the effects of release rates relative to the method and timing of augmentative biological control applications (Daane and Yokota 1997; McDougall and Mills 1997; Campbell and Lilley 1999; Clark et al. 2001; Jung et al. 2004). Two studies analyzed the impact of release rates when insecticides were used in conjunction with an augmentative biological control agent (Hough-Goldstein and Whalen 1993, van Driesche et al. 2001). For each study, the relative impact of release rates compared to these factors is discussed.

**Table 1b.**
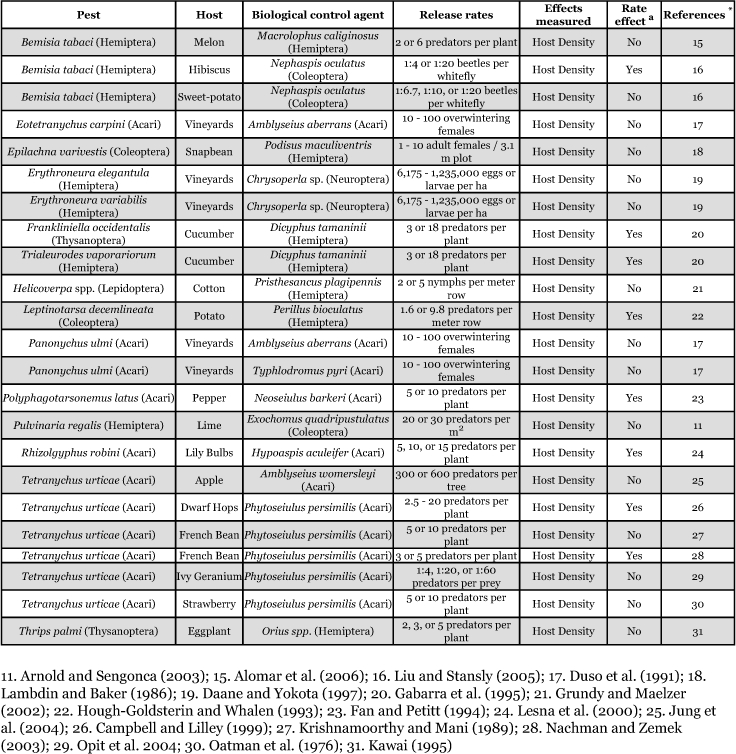
Impact of release rates of biological control agents on selected pests (Predators)

## Results

### Impact of release rate when parasitoids were used for biological control

**Figure 1.**
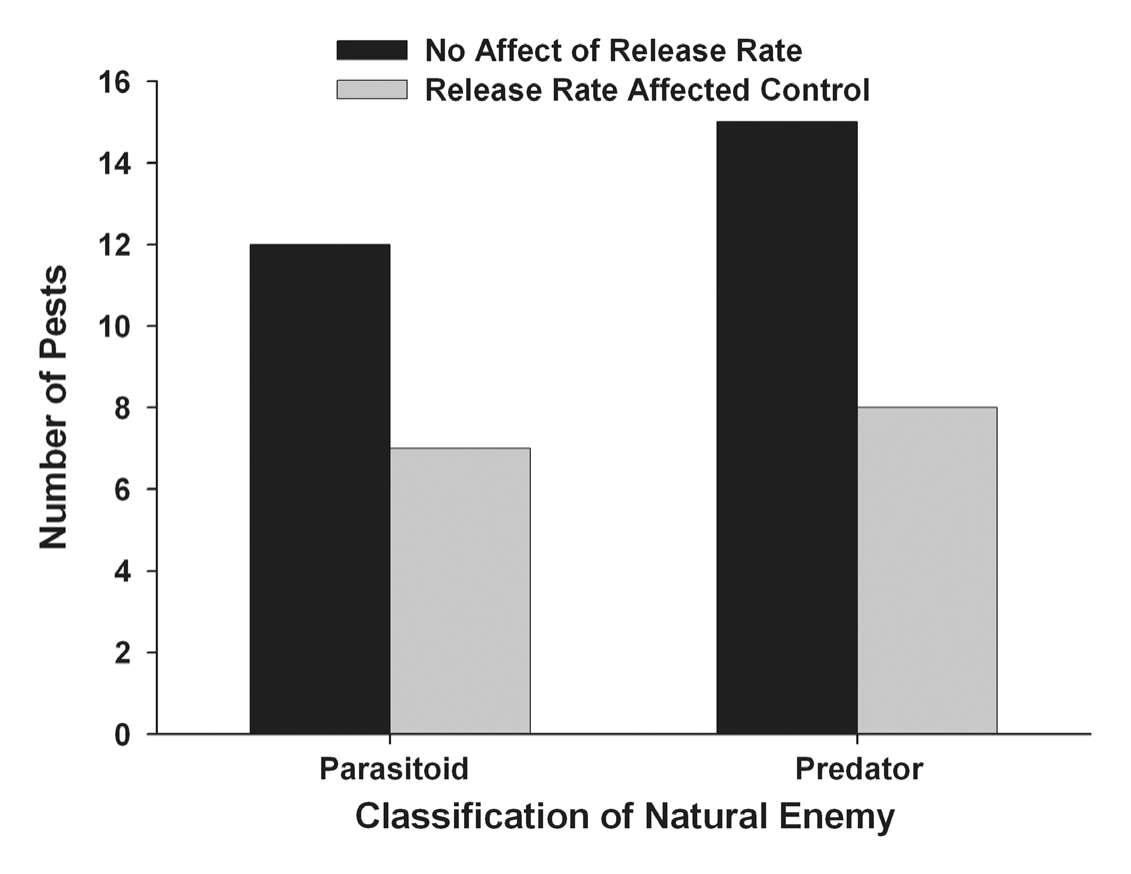
Number of pests affected by release rate with either a predator or a parasitoid as the natural enemy utilized for augmentation biological control.

**Figure 2.**
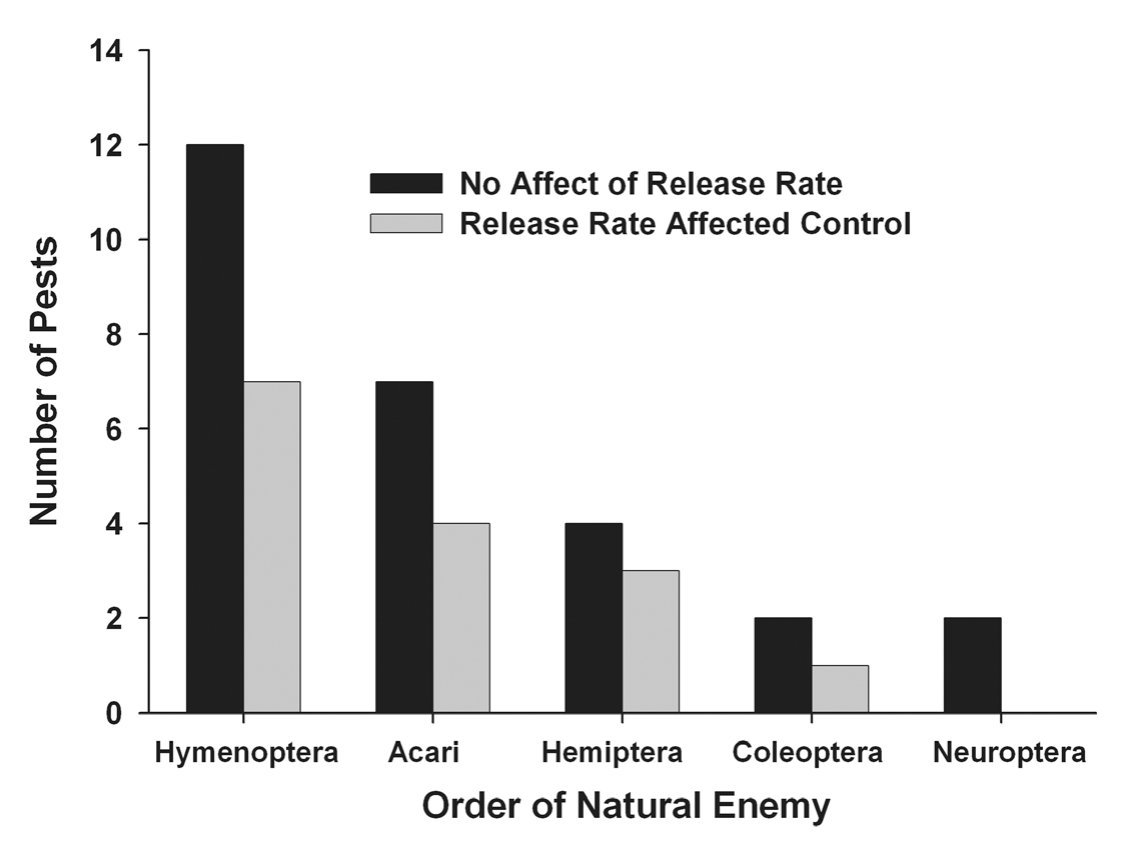
Number of pests affected by release rate with various orders of natural enemies utilized for augmentation biological control.

Of the 19 pests targeted with a parasitoid, in 12 cases (63%), increasing the release rate of the parasitoid did not significantly affect the density or mortality of the pest or the rate of parasitism (Del Bene 1990; Peterson and Cawthra 1995; Hoddle et al. 1997, 1998; Fernandes et al. 1998; Hommay et al. 2002; Arnold and Sengonca 2003; Stansly et al. 2005; Bellows Jr. et al. 2006) (Table 1, Figure 1). All of the parasitoids used were Hymenoptera. Thus, release rates of Hymenopteran parasitoids did not significantly impact the effectiveness of biological control in 63% of studies reviewed (Table 1, Figure 2).

Unlike the studies involving predators described later, in the 7 cases where release rates did affect augmentative biological control with a parasitoid, the highest release rate tested in each study did not reduce pest densities to near O (Wen and Grower 1994; Peterson et al. 1995; Norton and Welter 1996; McDougall and Mills 1997; Hommay et al. 2002; Stansly et al. 2005; Li et al. 2006). Thus, in these studies, an optimal release rate threshold where further increases were unlikely to increase control could not be identified. This result may be attributable to the observation that reductions in pest abundance are typically much greater when predators are used as augmentative biological control agents compared to parasitoids (Stilingand Cornelissen 2005).

### Impact of release rate when predators were used for biological control

Of the 23 pests targeted with a predator, in 15 cases (65%), increasing the release rate of the predator did not significantly affect the target pest density (Oatman et al. 1976; Krishnamoorthy and Mani 1989; Duso *et al.* 1991; Kawai 1995; Lambdin and Baker 1996; Daane and Yokota 1997; Grundy and Maelzer 2002; Arnold and Sengonca 2003; Jung et al. 2004; Opit et al. 2004; Liu and Stansly 2005; Alomar et al. 2006) (Table 1, Figure 1).

Of the 11 pests managed with predatory mites (Acari), in 7 cases release rates did not impact control of the pest (Oatman et al. 1976; Krishnamoorthy and Mani 1989; Duso *et al.* 1991; Fan and Petitt 1994; Campbell and Lilley 1999; Lesna et al. 2000; Nachman and Zemek 2003; Jung et al. 2004; Opit et al. 2004). Of the 7 pests managed with Hemipteran predators, 4 were not affected by release rate and 3 were affected by release rate (Lambdin and Baker 1986; Hough-Goldstein and Whalen 1993; Gabarra et al. 1995; Kawai 1995; Grundy and Maelzer 2002; Alomar et al. 2006) (Table 1, Figure 2). Two pests managed with a Coleopteran were affected by release rate and one was not (Arnold and Sengonca 2003; Liu and Stansly 2005) (Table 1, Figure 2). Both pests managed with Neuropterans were not affected by release rate (Daane and Yokota 1997).

In all 8 cases where release rates of predators affected augmentative biological control (Table 1), the author(s) demonstrated a threshold where further increases in release rates would not have improved control. For example, control of the sweetpotato whitefly, *Bemisia tabaci* (Gennadius), on hibiscus with the lady beetle, *Nephaspis oculatus* (Blatchley), with the highest release ratio of 1:4 beetle: whitefly resulted in no visible damage to any hibiscus plants, indicating that higher release rates would not have improved whitefly control. In another study, elimination of the bulb mite, *Rhizoglyphus robini* (Claparede), with releases of a predatory mite, *Hypoaspis aculeifer* (Canestrini), was possible with a release rate of 3:1 predators:prey in field and greenhouse experiments but not with lower rates (Lesna et al. 2000). Results in the other six cases were similar, as in each study the highest release rate tested reduced pest populations, or damage, to negligible levels, indicating that further increases would not have significantly increased control (Hough-Goldstein and Whalen 1993; Fan and Petitt 1994; Gabarra et al. 1995; Campbell and Lilley 1999; Lesna et al. 2000; Nachman and Zemek 2003).

### Impact of release rate on targeted pests

**Figures 3.**
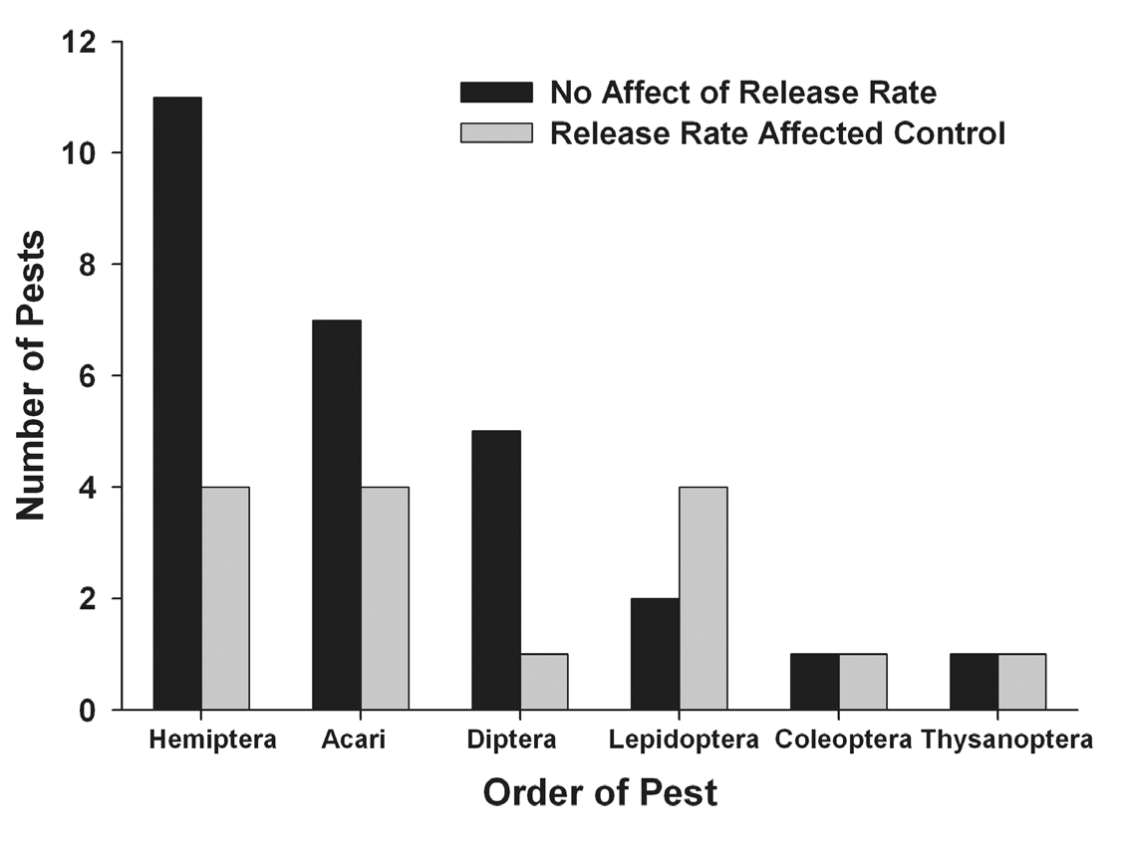
Number of pests affected by release rate with various orders of pest targeted for augmentation biological control. Pests were grouped by order regardless of the type of natural enemy (parasitoid or predator).

Hemiptera was the most common pest order, regardless of whether the biological control agent was a parasitoid or predator (15 pests, 36%), followed by Acari (11 pests, 26%), Diptera (6 pests, 14%), Lepidoptera (6 pests, 14%), Coleoptera (2 pests, 5%), and Thysanoptera (2 pests, 5%) (Table 1, Figure 3). The control of Hemipteran pests was not affected by release rate in 11 out of 15 cases. The control of mites (Acari) was not affected by release rate in 7 of 11 cases. In 5 out of 6 cases the control of Dipteran pests was not affected by release rate, while the control of Lepidopteran pests was not affected by release rate in only 2 out of 6 cases. In 1 out of 2 cases the control of either Coleopteran or Thysanopteran pests was not affected by release rate (Table 1, Figure 3).

### Impact of release rate relative to the method and timing of application

In some cases, the release rate of a biological control agent had a relatively small impact on the control of a pest compared to the method and timing of application of the agent. The method of application primarily affects the ability of a biological control agent to establish in the field, which is necessary for long-term control of a pest. The timing of release of a biological control agent affects its synchrony with the host insect, which can improve the chances of success. Several studies compared the effects of release rates relative to the method and timing of biological control applications (Daane and Yokota 1997; McDougall and Mills 1997; Campbell and Lilley 1999; Clark et al. 2001; Jung et al. 2004).

For example, augmentative biological control of two leafhopper pests, *Erythroneura variabilis* (Beamer) and *E. elegantula* (Osborn), in vineyards with green lacewings, *Chrysoperla* spp., was not affected by release rates but was affected by the method and timing of application (Daane and Yokota 1997). Releases of green lacewings at densities from 6,175 to 1,235,000 eggs or larvae per ha provided similar levels of control. However, releases that were timed to approximately 50-70% leaf hopper egg hatch had a greater effect on densities than releases timed to peak leaf hopper nymphal densities. In addition, releases of green lacewing larvae were more effective than releases of lacewing eggs (Daane and Yokota 1997). In another study, releases of the predatory mite *Phytoseiulus persimilis* (Athias-Henriot) early in the season to control the two-spotted spider mite, *Tetranychus urticae* (Koch), on dwarf hops maintained populations at lower densities than releases later in the year regardless of the release rate (Campbell and Lilley 1999).

In another study, when the predatory mite *Amblyseius womersleyi* (Schicha) was used to control populations of the two-spotted spider mite, *Tetranychus urticae,* initial settlement of the predatory mites, which was aided by multiple releases, was significantly more important than release rate in determining the effectiveness of control (Jung et al. 2004). For control of spotted knapweed, *Centaurea maculosa* (Lamarck), many, smaller releases of two herbivores, *Cyphocleonus achates* (Fahraeus) and *Agapeta zoegana* (L.), was more effective than fewer, larger releases, while release rate did not affect establishment of the herbivores (Clark et al. 2001). Similarly, many releases of the egg parasitoid *Trichogramma* spp. were necessary for control of the codling moth, *Cydia pomonella* (L.), regardless of release rate (McDougall and Mills 1997).

### Impact of release rate with insecticide usage

Two studies reviewed analyzed the impact of release rates when insecticides were used in conjunction with a biological control agent. When the parasitoid *Eretmocerus eremicus* (Rose and Zolnerowich) was used in combination with the insect growth regulator buprofezin to control two whitefly species, *Trialeurodes vaporariourum* (Westwood) and *Bemisia argentifolii* (Bellows and Perring), lower release rates provided effective control when used in combination with insecticides (van Driesche et al. 2001). A release rate of one parasitoid per plant per week in combination with a mid-season application of buprofezin reduced pest densities as effectively as releasing 3 parasitoids per plant per week without insecticides or 2 parasitoids per plant per week with a mid-season application of buprofezin. The low release treatment with buprofezin cost an average of $0.38 per plant, while the other two treatments cost $1.18 and $0.75, respectively, indicating that using a low release rate along with insecticides was most beneficial from an economic perspective (van Driesche et al. 2001).

As mentioned earlier, the Colorado potato beetle was managed more effectively with high release rates of predatory stink bugs than low release rates (Hough-Goldstein and Whalen 1993). However, when a low release rate was combined with an application of the toxin *Bacillus thuringiensis* (Bt), the level of control achieved was not significantly different from that with a high release rate and was significantly greater than either a low release rate alone or Bt alone (Hough-Goldstein and Whalen 1993). The results of these two studies indicate that lower release rates of biological control agents are often as effective as higher rates when biological control is used in conjunction with insecticides.

## Discussion

The effectiveness of augmentative biological control agents for controlling arthropod pests was not significantly affected by the release rate in 64% of the cases reviewed. Results were similar when comparing studies that utilized parasitoids as biological control agents (63%) with studies that utilized predators (65%). With any order of natural enemy, there were more cases where release rates did not affect augmentative biological control than cases where release rates were significant (Table 1, Figure 2). There were more cases where release rates did not affect augmentative biological control when pests were from the orders Hemiptera, Acari, or Diptera, but not with pests from the order Lepidoptera (Table 1, Figure 3). These results demonstrate that the relative impact of release rates on the success of augmentative biological control may be affected by both the order of the natural enemy and the pest. Other factors, such as the cropping system, may also be significant in determining the impact of release rates.

Increasing release rates may not increase the effectiveness of augmentative biological control for several reasons. If lower release rates provide enough natural enemies to completely eliminate or significantly reduce pest populations, increases in the rate may only result in higher mortality of the biological control agent. In addition, biological control agents that can successfully establish in an area and have a high reproductive potential may be effective at lower rates because they can efficiently grow and reproduce in the field (Petersen and Cawthra 1995; Hoddle et al. 1997, 1998).

This study identified 8 factors that limited the relative impact of release rates on the effectiveness of augmentative biological control: prey availability, initial settlement rates, fecundity, dispersal, cannibalism, the method of release, the timing of releases, and insecticides. First, as release rates increase, the ratio of the number of prey per natural enemy decreases. Thus, although higher release rates increase the number of natural enemies in an environment, fewer prey may be attacked by each natural enemy. If fewer natural enemies are able to affect the same number of prey as larger numbers, release rates become less significant (Duso *et al.* 1991; Petersen and Cawthra 1995; Alomar et al. 2006; Bellows Jr. et al. 2006). Second, in some cases settlement rates of natural enemies were similar when comparing high and low release rate treatments (Jung et al. 2004; Alomar et al. 2006). Density-dependent survival and other factors can result in greater mortality of natural enemies at high release rates, which can ultimately result in the same number of natural enemies settling in an area regardless of release rate (Jung et al. 2004; Alomar et al. 2006). Third, in several cases the fecundity of natural enemies increased at lower release rates (Petersen and Cawthra 1995; Hoddle et al. 1998; Alomar et al. 2006). This can result in similar population densities of natural enemies over time in high and low release rate treatments. Fourth, density-dependent dispersal of natural enemies may occur at higher rates with high release densities, resulting in similar population densities compared to low release treatments in the target area (Grundy and Maelzer 2002). Fifth, in one case reviewed, release of green lacewings larvae at high densities cannibalized each other at high rates, resulting in similar population densities compared to low release rate treatments (Daane and Yokota 1997). Collier and van Steenwyk (2004) showed that 12 ecological factors could potentially limit the efficacy of augmentation biological control. Results in this review demonstrate that similar ecological factors may limit the impact of release rates on augmentation biological control.

In some cases, lower release rates may actually provide better control than higher rates (Hoddle et al. 1997). One mechanism by which lower release rates might be more effective is through mutual interference. This occurs when parasites or predators that are searching for a host encounter each other, which can cause one or both to stop searching and possibly leave the area (Hassell and Varley 1969). Actual contact is not necessary as recognizing a cue left by another natural enemy can be as effective. The effects of mutual interference can be magnified as biological control agent densities increase, which can occur with higher release rates.

The timing and method of biological control applications often were more significant factors affecting the success of biological control than the release rate (Daane and Yokota 1997; McDougall and Mills 1997; Clark et al. 2001; Jung et al. 2004). In their review of augmentative biological control, Collier and van Steenwyk (2004) suggested that the use of insecticides may be an important factor that can improve the effectiveness and economical use of augmentative biological control. As discussed above, lower release rates were often optimal when biological control was used in conjunction with insecticides (Hough-Goldstein and Whalen 1993; van Driesche et al. 2001).

The studies discussed here suggest that for most augmentative biological control agents, there is an optimal release rate that produces effective control of a pest species. This was especially true when predators were used in biological control. Increasing the release rate above the optimal rate does not improve the control of pest species and is potentially economically detrimental. The optimal release rate of a biological control agent may depend on the host crop (Liu and Stansly 2005; Stansly et al. 2005).

It is clear that release rates are a factor that should be carefully considered before implementing any augmentative biological control effort. This review showed that, in most cases, increasing the number of natural enemies released did not necessarily increase the effectiveness of augmentative biological control. The ultimate success of augmentative biological control may depend on releases of biological control agents that maximize establishment, are released in synchrony with the host, and can be integrated into integrated pest management programs in conjunction with insecticides. Thus, determining optimal release rates that maximize the effectiveness of natural enemies can increase the effectiveness of augmentation biological control and increase its potential economic benefits.
